# Correlational analysis of distinct contributions and overlaps between visual, visual attention, and perceptual spans

**DOI:** 10.1038/s41598-026-38243-x

**Published:** 2026-02-12

**Authors:** Aline Frey, David Meary, Murielle Loichot, Jonathan Mirault, Marie-Line Bosse

**Affiliations:** 1 Aix-Marseille Univ, CNRS, Marseille, CRPN France; 2 Univ. Grenoble Alpes, Univ. Savoie Mont Blanc, CNRS, Grenoble, 38000 LPNC France; 3 National Education, Annecy, France

**Keywords:** Neuroscience, Psychology, Psychology

## Abstract

The visual span, the visual attention span and the perceptual span are defined within specific theoretical frameworks. Each framework uses different measurement methods that reflect specific visual processes. All three are commonly associated with reading skills, and they are often confused or overlapped in the literature. For the first time, these three spans have been assessed in the same participants and under the same methodological conditions, enabling a more accurate comparison to be made. Correlations between the spans and with control tasks confirm certain peculiarities but also reveal some similarities. Reading ability correlates significantly with each span. However, only the perceptual span explains a specific part of the variance in adult reading speed.

## Introduction

 It is widely recognized that reading engages motor, perceptual, and cognitive faculties. Of particular importance is the role of visual processing, which can create a bottleneck effect known as span in the literature. Within specific theoretical frameworks, three kinds of spans are traditionally defined^1^ : the visual span (VS), the visual attention span (VAS), and the perceptual span (PS). Each has distinct measurement methods and reflects specific treatments.

An important remaining question concerning these three spans is how they are implicated in the reading process. Most studies have focused on the role of phonological and linguistic skills in reading. A widely accepted view is that these skills predict reading performance and account for differences in reading ability. Thus, it has been suggested that the ineffective eye movement control observed in less proficient readers is a consequence of linguistic processing difficulty^2,3^ rather than a visual or perceptual difference. However, the idea that reading skills are solely, or predominantly due to top-down linguistic influences has been debated elsewhere^4^, and the question remains as to the respective contributions of these different spans to reading. In the following sections of this introduction, we will describe each of these three spans, providing their definition, how they have been measured, their sizes, their respective bottom-up and/or top-down influences and, almost importantly, their potential relationship with reading ability.

VS for reading refers to the maximum number of letters, arranged horizontally as in a text, that can be accurately recognized without moving the eyes. It is typically assessed using the “trigram method,” in which participants are asked to identify letters, in trigrams of letters, flashed briefly at various positions to the left and right of a fixation point^5^. Through repeated trials, a VS profile is generated, depicting letter recognition accuracy as a function of distance from the fixation point in both directions. VS size is measured by the number of letters, to the left and right of the fixation point where letter recognition accuracy exceeds a predetermined threshold, often set at 80%. In adults with normal vision, the VS is approximately 10 characters (five characters can be recognized on either side of the fixation point, with over 80% accuracy). Recognition accuracy declines rapidly and monotonically for letters farther away from central fixation. Because the VS measure is based on the processing of single letters, it is expected to be relatively independent of top-down contextual influences, except for knowledge of letter names. It is mainly influenced by sensory factors such as text contrast, print size^6^, character spacing^7^, and retinal eccentricity^5^. The VS also refers to the crowding effect, which increases when trigrams are positioned away from the fovea. The VS captures two essential aspects of visual processing that are crucial for reading: identifying individual letters and encoding their relative positions. Thus, it has been proposed as a bottom-up sensory limitation on the number of letters that can be recognized without moving the eyes. This limitation is distinct from attentional, motor or linguistic influences^6^ and effectively imposes a limit on reading speed. The VS provides a theoretical framework for understanding how stimulus attributes, such as letter size, layout and contrast, impact reading speed. The influential role of VS size on reading speed has also been demonstrated in a computer model called “Mr. Chips”, which uses VS size as a key parameter^8,9^. This model provides evidence of a link between reading speed and VS size. From a developmental point of view, links have been shown between changes in VS size in school-age children and their reading speed. Kwon et al.^10^ demonstrated that VS size increases linearly with grade level and regression analysis revealed that 34–52% of variability in reading speed is explained by VS size. These findings are consistent with the significant role of early visual processing in developing reading skills.

The VAS is the number of orthographic units (e.g., letters, letter groups or syllables) that can be processed simultaneously at a single glance within a multi-element array during the first 200 ms^11–13^. The VAS is estimated through whole and partial letter report tasks^11^, in which consonant strings (e.g., R V S N T) are displayed briefly at the center of the computer screen. Participants are asked to orally report either all of the letters, regardless of their position (whole report paradigm), or a single post-cued letter (partial report paradigm). Standard VAS assessment also includes a control task measuring single letter identification threshold^14^. Adults reported on average five letters (5.2 during the global report condition, 4.9 during the partial report condition^15^). From a theoretical perspective, the VAS should essentially depend on the visual attention system responsible for the ability to simultaneously process letter strings, spaced far enough to avoid crowding, from serial to more parallel processing^13,16,17^. Several studies in adults have demonstrated a positive correlation between visual attention and reading time^18^. In some computational reading models, visual attention plays a key role in facilitating the shift from serial to parallel processing^16,17^, where words are processed as a whole. The extent of visual attention plays a crucial role in word processing, because when it does not cover the entire word, words cannot be processed holistically and are instead read through the serial activation of smaller orthographic units, such as syllables, letter clusters, or individual letters^19^. The links between VAS and reading skills have primarily been examined from a developmental point of view. Numerous studies have shown that visuo-attentional skills are involved in typical^20–22^ and impaired reading^23^. Valdois et al.^24^ explored the cognitive profiles of a population of 948 children identified as poor readers, to develop more effective intervention responses. They observed a VAS deficit in 18% of these poor readers (in a sub-sample of 110 children). It thus seems that the orientation of visual attention is significantly involved in the development of reading skills. In developmental dyslexia (DD), the VAS deficit hypothesis suggests that a subset of dyslexic individuals shows a multielement parallel processing deficit due to reduced visual attention capacity^14^. This hypothesis is in accordance with the idea that deficits in VAS lead to a cascade of effects in the visual coding of letters, including impairments in the visual processing of graphemes, their translation into phonemes and even the development of phonemic awareness^23^. More precisely, the VAS deficit appeared independently of the type of stimuli (verbal or visual), confirming the hypothesis of a visual attention span, as opposed to a verbal span deficit^25^. However, the question of the origin of reading difficulties in DD remains highly debated, particularly with regard to phonological deficits^26^. A recent meta-analysis^27^ of 25 studies confirmed the existence of VAS deficits in dyslexia, with a greater deficit in more opaque languages. What is more, dyslexic children who have been trained with VAS-related exercises improved their reading accuracy of silent sentences after training, suggesting far-transfer effects at the linguistic level^28^.

Finally, the PS^29^ refers to the region of visual field in which useful information is acquired in reading. The size of the PS has been estimated using an eye-tracking based technique, namely the moving window technique^29,30^, in which an area of text around each fixation is displayed normally, but text beyond this area is masked, by replacing the original letters with “x”. Using this technique, the PS size is estimated to extend about 14–15 characters to the right of fixation and 3–4 characters to the left of fixation, in English^29,31–33^. The PS is believed to reflect parafoveal preview, thus indicating linguistic or cognitive processing rather than low-level visual processing^2^, although some studies have shown that factors such as contrast influence reading speed^9^. However, actual measurement of PS does not allow distinguishing between the various perceptual and/or linguistic contributions^1^. Fixation times are largely modulated by the ‘foveal load’ of the current fixation; e.g., the duration of the first fixation and the duration of gaze on the upcoming word *N* + 1 (parafoveal) were significantly shorter when the current word N (foveal) was more frequent. The PS has also been extensively studied in relation to reading ability. Research suggests that readers’ PS is modulated by individual differences in language skills, with greater language proficiency associated with more efficient mechanisms for extracting linguistic information beyond the fixated word^34^. For instance, Veldre and Andrews^35^, in two experiments using the gaze-contingent moving window paradigm, showed that reading comprehension and spelling ability modulate the PS of expert adult readers. Participants with high reading and spelling skills benefited most from windows longer than 11 characters, primarily due to the increased length of forward saccades. They were also significantly more disturbed by the absence of nearby parafoveal information than those with low reading and/or spelling abilities. From a developmental perspective, studies indicate that PS size increases mainly during the early primary school grades and reaches adulthood levels at around sixth grade (e.g.,^2,36,37^).

In summary, all three spans are involved in reading skills in some way, whether during development or in adults with expert skills. However, it has not yet been determined which span is more involved than the others. This is one of the main objectives of this study. Furthermore, in the literature, there is frequent confusion or overlap between these different terms (VS, VAS and PS). For example, Grainger, Dufau and Ziegler^38^ have grouped these spans under the same concept: “span of effective vision for reading” (p. 172). In a previous review^1^, we attempted to clarify the common features and differences between these three concepts. However, to the best of our knowledge, these spans have always been studied independently, with different participants and under diverse experimental conditions. Factors such as letter size, letter font or contrast, for example, can vary from one experiment to another, which can have a significant influence on span measurement, particularly for the VS^6,39^. Therefore, the objective of this research is to evaluate these three spans for the first time in the same adult participants under identical methodological conditions. These assessments will help clarify the relationships between the three spans, and to determine their respective correlation with reading skills.

## Results

### Descriptive analyses: the different spans

Each span was estimated using a different paradigm, but with exactly the same letter features (see Method section). To make the spans comparable, they were all expressed in terms of the number of letters and degrees of visual angle. The datasets analyzed during the current study are available in the OSF repository, https://osf.io/kxqyg/.

### Visual span

VS was estimated using the trigram paradigm. It corresponds to the angular visual degrees (or the number of letters that can be written in this space) for which participants correctly identified the central letter of the trigram 80% of the time. The mean VS for our participants (*N* = 28, two participants had missing data due to technical issues) was 7.83 letters (SD = 1.71, range = 3.57–10.71). Participants identified slightly more letters to the right of the fixation point than to the left (4.1 vs. 3.73 letters, respectively). In degrees of visual angle, the mean VS was of 4.39° (SD = 0.96, range = 2–6), with 2.09° to the left and 2.3° to the right.

### Visual attention span

VAS was estimated using the average score from both the global report and partial report tasks. It corresponds to the average number of letters correctly reported among the 6 letters in each item. The mean VAS for our participants (*N* = 30) was 5.09 letters (SD = 0.42, range = 4.23–5.85), which is equivalent to 4.73° (SD = 0.39, range = 3.93–5.44) in degrees of visual angle.

### Perceptual span

PS was estimated using the gaze-contingent moving window paradigm. It corresponds to the size of the smallest window in which the reading speed was similar to that in the no-window condition. The mean perceptual span of our participants (*N* = 30) was 8.87 letters (SD = 3.54, range = 2–18) or 4.79° (SD = 1.91, range = 1.08–9.72) in visual angular degrees. Since eye movements were recorded during the PS task, we were able to analyze the evolution of saccades and fixations depending on window size. A t-test comparing each window size condition to the no-window condition revealed that all eye movement measures were affected in the smallest window condition (4 letters available to the left of the fixation and 3 to the right): the number of fixations and the duration of fixations increased (t(29) = −7.55, *p* <.001 and t(29) = 8.49, *p* <.001 respectively). The total number of progressive saccades per sentence increased (t(29) = −13.04, *p* <.001) while their amplitude decreased (t(29) = 13.59, *p* <.001). There were also fewer regressive saccades (t(29) = 3.37, *p* <.01). These characteristics remained consistent in the slightly wider window condition (4 letters available to the left of the fixation and 7 letters to the right), with a nearly significant result for the number and duration of the fixations (t(29) = −1.95 and t(29) = 1.93 respectively, both ps < 0.06). When the window size increased further, fixation time normalized. The number of fixations and saccades was slightly lower than in the no-window condition, though there were still fewer regressive saccades.

### The relations between the different spans

Table 1 shows the correlation between the three spans, each measured in terms of number of letters. The correlations between the VS and both the VAS and the PS spans were significant. The correlation between VAS and PS was not significant (p = .24). To better describe the skills on which these spans could depend, the correlations between each span and the control measures can be seen in Table 2. The three spans significantly correlate with verbal short-term memory. Only the VAS shows significant correlation with both the working memory and the focused attention score.

**Table 1:** Pearson correlations between the three spans.

Spans	VS	VAS	PS
VS	1	0.460**	0.464**
VAS		1	0.220
PS			1

Note: *N* = 28; ** = *p* <.01.

**Table 2:** Pearson correlations between the three spans and the control measures.

Spans	Focused attention	Short Term Memory	Working Memory	Letter identification
VS	0.167	0.317**	0.266	0.223
VAS	0.473**	0.426**	0.624***	0.109
PS	0.114	0.408*	0.141	0.116

Note: *N* = 28; * = *p* <.05; ** = *p* <.01; *** = *p* <.001.

**Table 3:** Pearson correlations between the three spans and measures obtained during normal sentence reading.

Spans	Reading time	Number of Fixations	Fixation duration	Number of pro-saccades	Amplitude of pro-saccades
VS	-.335*	-.392*	-.123	-.426**	.288
VAS	-.392*	-.408*	-.193	-.361*	.272
PS	-.473**	-.359*	-.458**	-.337*	.300

Note: N = 28; * = p < .05; ** = p < .01

### The relations between the different spans and sentence reading

**Fig. 1:**
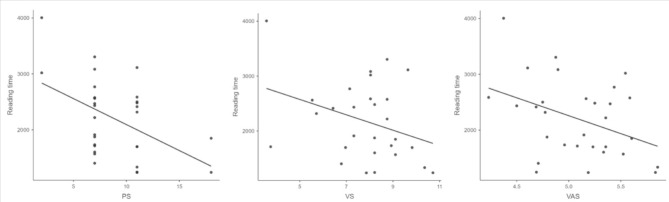
Scatter plots of correlations between different spans and reading time.

Table 3 shows the correlations between the three spans and various behavioral and eye-tracking measures obtained during normal sentence reading. As expected, the three spans are significantly and negatively correlated with reading time: the larger the span, the faster the reading speed (see Fig. 1). The three spans also significantly and negatively correlate with the number of fixations and the number of progressive saccades. Perceptual span is the only span that correlates with fixation duration: the larger the perceptual span, the shorter the fixation duration. No span significantly correlates with the progressive saccade length.

Finally, linear regressions were conducted on the reading measures for which significant correlations were found with the three spans: reading time, number of fixations and number of progressive saccades (see Table 4). The respective total R² were 0.315 (F(3, 24) = 3.681, *p* =.026), 0.260 (F(3,24) = 2.804, *p* =.061) and 0.249 (F(3,24) = 2.647, *p* =.072). The PS appears to account for a significant amount of the variance in reading time (∆ R² = 0.126), even when controlling for the contributions of the other spans. On the contrary, VS and VAS do not explain a specific part of variance in reading time. No single span explains a specific part of the variance in either the number of fixations or the number of saccades. Overall, little of the variance in oculomotor measurements (only 25%) is explained by the three spans combined.

**Table 4:** Results of the linear regression on reading time, number of fixations and number of pro-saccades, with the three spans included as independent variables.

Reading time	β	t	*p*
VS	− 0.015	−0.072	0.943
VAS	− 0.290	−1.522	0.141
PS	− 0.401	−2.102*	0.046
Number of fixations	*β*	t	p
VS	− 0.160	−0.741	0.466
VAS	− 0.267	−1.347	0.191
PS	− 0.236	−1.190	0.246
Number of pro-saccades	*β*	t	p
VS	− 0.245	−1.125	0.272
VAS	− 0.238	−1.191	0.245
PS	− 0.156	−0.778	0.444

## Discussion

The first objective of this study was to evaluate the VS, the VAS and the PS in the same adult participants and under the same methodological conditions, to more accurately describe their similarities and differences, as these terms are often mistaken for one another. All of these spans have been described as being related to reading. However, their relationship to reading has never been directly compared, which was the second objective of this study. First, since we have modified the methodology used to measure these three spans to standardize them, it is important to describe how these modifications may have affected their respective size.

In short, we can see that the size of the VS we observed (almost 8 letters) is slightly smaller than those reported in the literature (around 10 letters). For example, Risse^40^ obtained a mean visual angle of 10.9 letters. This was to be expected, given that our letters were wider (0.54°) than those used in other experiments (0.46°^40^). This confirms that the VS is size-dependent. Furthermore, like previous studies, our VS is slightly asymmetrical, larger toward the right, indicating a right visual field advantage for word-like stimuli^41^. We used the conventional VAS measurement methodology (except for the font), and we obtained the same span size, i.e., around 5 letters or 4.65°^15^. Finally, regarding PS, the mean PS of our participants was 8.87 letters or 4.79 degrees of visual angle. At first glance, this value may seem smaller than the estimates reported in the literature (see the Introduction). Some earlier studies suggest that the number of letters included in PS may depends on letter size. For example, McConkie and Rayner^42^ reported a PS of 19 letters, despite their study having only three participants and using early eye-tracking techniques. In contrast, Miellet, O’Donnell and Sereno^43^ found that enlarging parafoveal letters did not significantly change the PS when using a parafoveal magnification paradigm, indicating that attentional rather than purely visual constraints may limit the PS. Overall, our results seem to support the idea that the PS remains relatively stable in angular terms, while the number of letters it encompasses may vary depending on the experimental conditions. By homogenizing the presentation and measurement parameters of the spans (same letter size, font, etc.), we obtained results that differ from those classically observed in the literature, with a reduction in the VS size, especially that of PS. This is an important finding to consider for future studies that aim to compare the implications of these 3 spans.

Having therefore taken the precaution of homogenizing the measurements of the 3 spans, we can now compare them more accurately. Our results show that only the VS correlates with the VAS and PS, however the latter two do not correlate with each other. It is not surprising to find that the VS correlates with the other two spans since, as seen above, it is mainly sensitive to low-level characteristics, such as letter contrast, spacing and print size. These features are indeed found in the VAS and PS measurement tasks. We also observe that the three spans are significantly correlated with verbal short-term memory, perhaps because the tasks used for these three spans all require processing and oral pronunciation of items. Consequently, their correlation could also be interpreted as a shared reliance on short-term memory.

Interestingly, VAS and PS do not correlate. The VAS is considered a measure of visual attention for the parallel processing of letters, and appears relatively independent of the PS. The PS is considered to primarily reflect linguistic abilities^34,44^ and the parafoveal processing of words in the sentence^43^. The independence of these two spans can be explained by at least two main differences between them. First, attentional skills appear to be involved differently in each span. While the VAS assesses the ability to extend visual attention over a set of letters in the foveal zone without shifting attention, the PS assesses the ability to process information in the parafoveal zone and shift attention. Accordingly, the VAS is the only span related to the D2-R test of focused attention^45^.

Secondly, PS relies heavily on linguistic skills of reading and recognizing written words. In contrast, VAS requires minimal language skills, only the knowledge of letter names. Studies on dyslexia typically find VAS in children with no linguistic deficits, such as a phonological processing deficit^11,14^, suggesting that VAS and phonological processing are two independent cognitive underpinnings of reading ability.

In accordance with the literature^6,18,35^, correlational analyses confirm that all three spans are involved in the reading process. All three correlate with reading speed, number of fixations, and number of progressive saccades. The larger the span, the faster the reader, with fewer fixations and saccades. However, fixation duration only correlates with PS: the higher the PS, the shorter the fixation duration. It’s as if people with high PS can process words faster while programming the next eye saccade. It is important to remember, however, that correlations do not indicate causality. Since many studies show that span size increases with age and reading level^10,36,46^, it’s also possible that span size is a consequence of reading level rather than its cause.

Linear regressions were performed to determine whether the different spans independently explained a significant portion of the variance in reading performance. Since those analyses were performed on only thirty participants, the results should be interpreted with caution. However, the results first suggest that the three spans together explain no more than one-quarter of the variance in oculometric reading data, and one-third of the variance in reading speed. Reading is a complex task requiring visual and phonological processing skills, as well as linguistic knowledge. These factors also play an important role in reading performance and speed. Second, the regression analyses show that neither the VS nor the VAS independently explains part of the variance in reading performance. Although they are supposed to measure different aspects of visual processing using distinct paradigms, it is challenging to identify the unique contribution of each span to reading performance. Ultimately, the results confirm that PS is the only factor that specifically contributes to reading speed. PS is modulated by both visual processing and language skills useful for extracting linguistic information^34^. This pattern of results aligns with prior research suggesting that the PS may be more closely linked to linguistic processing than VS or VAS^34,35^. However, since the study did not include direct measures of linguistic skills *(e.g.*., phonological awareness, vocabulary and reading fluency*)*, this interpretation should be considered a hypothesis based on prior research rather than an empirically validated conclusion from the present data. Further investigation is warranted.

However, several limitations of the present study should be acknowledged. First, our measure of reading speed was derived from the FL condition of the PS task. This raises the possibility of shared variance due to methodological overlap. Therefore, future studies should use independent reading tasks to better assess the PS’s unique contribution. Second, PS was measured as a discrete variable with fixed window sizes, following the classical moving-window paradigm. While this approach allows for comparison with existing literature, it limits precision. Continuous or adaptive measures could provide a more detailed assessment in the future. Nonlinear asymptotic modeling of reading speed as a function of window size^36^ offers a finer estimation of the perceptual span and could overcome some current methodological constraints. Third, although the set of sentences was carefully checked for length and difficulty, some sentences may be more difficult to read than others, due to longer words, for example. Additionally, the random assignment of sentences to the window size condition may have introduced random noise. A fixed or counterbalanced design could provide tighter control of sentence difficulty in future correlational studies. Another limitation of the present study is the lack of direct assessment of linguistic abilities. This prevents us from empirically testing the extent to which linguistic skills contribute to the observed relationships between the PS and reading performance. Future studies should include such measures to clarify the specific cognitive and linguistic determinants of each span. Finally, our sample size was relatively small and restricted to psychology students. To examine how these spans relate to each other across different stages of reading acquisition and expertise, larger and more diverse samples are necessary, including developmental populations.

Despite these limitations, our study shows that VS, VAS and PS all reflect different aspects of visual and linguistic processing. While VS correlates with both VAS and PS, only PS contributes specifically to reading speed. These findings underscore the importance of exercising caution when using and interpreting these spans, and highlight the need for future studies to confirm and clarify their specific roles in reading.

## Methods

### Participants

Thirty adult psychology students from Grenoble University (23 of whom were women) took part in the experiment. Their average age was 21 years and 10 months (SD = 4 years; from 17 to 33 years old). All participants were native French speakers, with normal uncorrected near and distance visual acuity^47,48^ and no detected reading disabilities. They all gave written informed consent to take part in the experiment.

### Material

#### Span tasks

The three span tasks were implemented using Matlab^®^ (R2007b) and the Eyelink^®^ toolbox from PsychToolbox^49^. To ensure comparability, all tasks used lowercase black letters in the Courier New Bold font at 15 points, on a white background.

Each letter had an angular size of 0.54 degrees when the participant’s eyes were 60 cm from the screen. Only the spacing between letters differed between tasks, which was adjusted to match the characteristics of each paradigm. In all the span tasks, stimuli appeared when the participant fixated on the fixation point for an average of 700ms (randomly +/−200ms, to limit anticipation phenomena).

Eye movements were recorded during the three span tasks, from a single eye via an Eyelink 1000^®^ (SR Research, Ltd., Ontario, Canada) at a sampling rate of 500 Hz. Stimuli were presented on a color monitor (Liyama Vision Master Pro 514, 40.5 × 30 cm) with a resolution of 1024 × 768 pixels and a refresh rate of 100 Hz. Participants were seated facing the screen, 60 cm away, with their head held fixed by a forehead rest. The Eyelink 1000^®^ eye-tracker was calibrated at the start of each task using a nine-point grid, followed by a nine-point validation.

• Visual span task.

A trigram paradigm was developed to estimate the VS. Each item was a randomly generated letter trigram presented for 100 ms with a letter spacing of 0.04°. Items appeared immediately after the central fixation point, ranging in eccentricity from 1° to 7° to the left or right. After the 10-item training phase, the 140-item test phase consisted of 10 items at each of these positions. Participants were asked to verbally report the central letter of each trigram. The VS was estimated using the Risse^40^ method, suggested by Legge et al.^5^. Identification accuracies for trigram locations were fitted for each participant using an asymmetric or split Gaussian function. Identification probabilities P(x) at different eccentricities (x) were given by the following equations: P(x)= Aexp (-x²/2*σ*²L), if x < 0 Aexp (-x²/2*σ*²R), if x > 0 where A, *σ*L and *σ*R denote the amplitude and the standard deviations of the left and right Gaussian curves, respectively. These three parameters were calculated for each participant based on their performance. The amplitude (A) is a probability value ranging from 0 to 1 that indicates the theoretical peak performance at the fixation position. The standard deviations can be considered as indicators of parafoveal processing (cf. however Risse^40^. From the Gaussian curve thus defined for each participant, we estimated the size of their VS, which corresponds to 80% correct identification. The VS can be expressed in degrees of visual angle or converted into a number of letters.

• Visual attention span tasks.

VAS was estimated based on both whole and partial letter report tasks^15^, using the following twelve consonants: b, c, d, f, h, l, n, p, r, s, t, v.

Whole report task: Twenty-four 6-letter strings were created, so that each consonant appeared ten times (two times in each position). No item included the same letter twice or a frequent bigram in French. After a fixation point and a 50 ms white screen, each item was presented in the central position for 200 ms, with a letter spacing of 0.47° and a total angular size of 5.6°. Participants completed ten training items before the 24 target items. They were asked to report all the letters they could remember, in no particular order and without time constraints. Their score was the total number of letters they correctly reported.

Partial report task: A total of sixty-two 6-letter strings were created, with the same characteristics as the previous task items. Each consonant appeared 30 times (six times in each position). After a fixation point and a 50 ms white screen, the item was presented in the central position for 200 ms. Immediately following this, a 50-millisecond visual cue consisting of a 5 mm vertical line was presented under the location of one of the six letters. Participants completed ten training items before the 72 target items. They were asked to report the cued letter aloud. Their score was the total number of letters they correctly reported. To obtain a single measure of visual attention span, the average score from the two tasks was converted into the mean number of letters per item (with a maximum score of six letters per item) or the corresponding degrees of visual angle.

• Perceptual span task.

We developed a sentence reading paradigm to estimate the PS and normal sentence reading speed. As previous princeps studies^29,50^, we used a gaze-contingent moving window paradigm in which the amount of information provided to the reader at each fixation point is controlled and varies across five conditions. There were four window conditions (4 letters are available to the left of the fixation point and 3, 7, 11 or 15 letters are available to the right of the fixation point; the other letters are replaced by X) and one full-length condition (FL condition; all the letters are available during stimulus processing). After the training phase which contained 10 sentences (2 for each window condition), the test phase consisted of reading 100 sentences, 20 sentences for each window condition. Each sentence contained between 7 and 14 words and between 57 and 60 characters, including spaces between words, with a character spacing of 0.04° and no punctuation marks except a final period. Their length allowed them to be presented on a single line, corresponding to a maximum angular size of 34°. The 100 sentences were adapted from royalty-free French literature^51^. Each sentence was randomly assigned to one of the five window conditions for each participant. The fixation point was positioned at the location of the first word of each sentence, on the left side of the screen. When the sentence appeared, participants had to read it silently and as naturally as possible. Then, they had to look at a gray square located at the bottom center of the screen, below the sentence. The sentence disappeared when the gray square was fixated on. To control the reader’s attention, 36% of the sentences were followed by a yes/no comprehension question. Eye-tracking data were processed in order to estimate both the perceptual span and the normal sentence reading speed. First, ‘saccade’ events were automatically defined by the Eyelink 1000^®^, according to predefined default saccade parameters, as a function of acceleration (> 8000°/sec²), speed (> 30°/sec) and position variation (> 0.15° variation). All non-saccadic events were considered as ‘fixation’ events. All events occurring before the final saccade toward the gray square were considered sentence-reading events. The reading area was defined as an 80-pixel-high rectangle located on the sentence (including 44 pixels below the bottom line of the letters). Events outside the reading area were deleted. The first fixation, which triggered the appearance of the sentence, was also excluded. For each sentence reading, the reading time was defined as the sum of all remaining fixations. Each participant’s normal sentence reading speed was calculated as the average reading time for the 20 sentences read in the FL condition. To estimate each participant’s PS size, we compared their reading times in each window condition with their reading times in the FL condition using t-tests. The PS size corresponds to the smallest window in which the reading speed was similar to that in the FL condition.

### Control tasks

- Near acuity.

Near acuity was tested using the Parinaud test^47^. We used the standard lettered model, which consists of a white PVC 1000 g/m² board with a handle and has dimensions of 221 × 135. The scale consisted of a series of texts, which were grouped into paragraphs of decreasing size. Participants held the board by its handle at a distance of about 33 cm (the normal reading distance) stretching out his arm. They began by reading paragraph 4 in a medium font. First, he read with both eyes. Then, we instructed him to read successively with one eye at a time, covering the other eye with a blindfold. We proceeded in this way for the following paragraphs. We determined the near visual acuity of each eye by identifying the paragraph with the smallest characters that the participant could read fluently without slowing down or changing the distance. Near acuity was expressed as a number corresponding to the size of the smallest readable characters (the greater the number, the poorer the acuity). A participant with a near acuity of 2 has normal near vision.

- Distance acuity.

Distance vision was tested using the Snellen scale^48^. The scale consists of a panel measuring 28 by 56 cm, with letters of different sizes arranged from largest to smallest from top to bottom. Participants stood six meters away the scale and had to read the letters one eye at a time, starting with the largest. The test stopped as soon as a mistake was made. For each participant, the visual acuity of each eye was expressed as a decimal (^52^ for details). A person with normal vision has a visual acuity of 1.0.

- Attention test D2R.

The D2-R is a test of focused attention^45^. Participants must cross out as many target letters as possible (a “d” with two dashes) from a number of distractors. The test consists of 14 lines, each containing a mix of target and distractor letters. Every 20 s, participants must move to the next line. The focused attention score is calculated using the number of targets processed and the error rate. Each participant’s score is standardized according to their age.

- Memory: WAIS span task (forward, backward and crescent).

Short-term and working memory abilities were estimated using three tasks from the number memory subtest of the WAIS-IV scale^53^. Participants had to orally report a series of digits spoken by the experimenter at a rate of one digit per second. In the first task, participants reported the series in the forward direction. In the second task, the reported the series in the reverse direction. In the third task, they reported the series in a crescent pattern. Each task comprised 16 series of items of increasing size. The task began with one training item of two digits, followed by two series of two digits, then two series of three digits and so on. The short-term memory score was the total number of items correctly reported in the forward series task. The working memory score was the average number of items correctly reported in the backward and crescent series tasks.

- Single-letter identification.

The single-letter identification threshold control task is typically used in conjunction with visual attention span assessments to control processing speed for single letter. Ten target letters were randomly chosen from the 12 letters used in the visual attention tasks. There were 50 target trials, with each letter presented five times at one of the following times: 33, 50, 67, 84 and 101 ms. The physical characteristics of the letters were the same as in the span tasks. The task began with ten training items (two for each presentation time) made with different letters from the ten target letters. Each trial began with a fixation point, followed immediately by the item in the central position. Then a mask (13 mm high and 37 mm wide) appeared for 150 ms. Participants were asked to orally report the letter they saw. As in previous VAS studies^46^, the number of correctly identified letters was weighted, so that the shortest presentation duration received the highest weight (the weighting was 5 for 33, 4 for 50, 3 for 67, 2 for 84 and 1 for 101 ms). The total score was the sum of all the weighted scores.

### Procedure

On average, all participants completed the tasks in a single 75-minute session. Half of the participants began with the span tasks, while the other half started with the control tasks. Within each group, the order of the tasks was randomized.

## Data Availability

The datasets analyzed during the current study are available in the OSF repository, https://osf.io/kxqyg/.
